# The application of pre-discharge delta total bilirubin to predict the need for post-discharge phototherapy in healthy neonates 35 weeks’ gestation or later

**DOI:** 10.1186/s12887-025-06238-8

**Published:** 2025-11-14

**Authors:** Thawinee Maneesilasan, Mallika Pomrop

**Affiliations:** 1 Department of Pediatrics, Faculty of Medicine, Chiang Mai University, Inthawarorod Road, Chiang Mai, Thailand; 2 Division of Neonatology, Department of Pediatrics, Faculty of Medicine, Chiang Mai University, Inthawarorod Road, Chiang Mai, Thailand

**Keywords:** Neonatal jaundice, Post-discharge phototherapy, New phototherapy guideline 2022, Pre-discharge delta total bilirubin

## Abstract

**Background:**

The American Academy of Pediatrics recommended the use of pre-discharge delta total bilirubin (DeltaTB) to determine appropriate follow-up timing.

**Objectives:**

To determine the proportion of neonates requiring post-discharge phototherapy after applying the pre-discharge DeltaTB approach, and to assess its effectiveness in predicting phototherapy needs.

**Methods:**

This is a prospective cohort study conducted at Chiang Mai University Hospital. The pre-discharge DeltaTB, defined as the difference between the bilirubin level and the phototherapy threshold at the time of measurement. Based on pre-discharge DeltaTB (mg/dL), patients were categorized into 3 risk groups: high-risk (< 3.5), moderate risk (3.5–6.9) and low risk (> 7). Post-discharge phototherapy, pre-discharge TB, and number of follow-up were compared between the risk groups. DeltaTB levels in different age intervals were analyzed using ROC curve analysis to determine the cutoff in each interval.

**Results:**

Out of 150 neonates, 31 (20.6%) required post-discharge phototherapy, with 9 (6.0%) neonates were classified as requiring subthreshold phototherapy. There were 17.3%, 53.3% and 19.3% of 150 neonates categorized as high, moderate, and low risk by pre-discharge DeltaTB, respectively. The high-risk group, had the highest proportion of post-discharge phototherapy, mean pre-discharge bilirubin level and number of follow-up visits and were significantly different from other groups (*P-value* < 0.001). The optimal timing for measuring pre-discharge bilirubin was aged 49–72 h (AUC of 0.851).

**Conclusion:**

Pre-discharge DeltaTB effectively stratified neonates by risk for post-discharge phototherapy and may guide timely follow-up to prevent severe hyperbilirubinemia, with significantly higher rates in the high-risk group.

## Introduction

Neonatal jaundice is one of the most common conditions in neonates [1–7]. affecting around 60% of term neonates and 80% of preterm neonates during their first week of life [1, 2, 8]. Pathological jaundice can progress to severe hyperbilirubinemia, which may lead to acute and chronic bilirubin encephalopathy, an irreversible complication [4]. Risk factors for neonatal jaundice can be categorized into maternal and neonatal factors. Maternal factors include Asian race, advanced maternal age, blood group incompatibility, lower gestational age, a family history suggesting hereditary red blood cell diseases, and gestational diabetes mellitus (GDM). Neonatal factors include male gender, low birth weight, scalp hematomas or bruising related to birth trauma, and exclusive breastfeeding with suboptimal intake [1, 2, 7, 9–12].

Most neonates develop jaundice within the first week of life [1, 6], with bilirubin levels typically peaking at approximately 72–120 h of age [6, 8]. In Thailand, healthy neonates are typically discharged at 2–3 days after birth. This is usually before their serum bilirubin reach to the peak levels. Therefore, neonatal jaundice is the leading cause of readmission in healthy neonates [1].

Delayed or inappropriate follow-up to newborn jaundice increases the risk of severe hyperbilirubinemia and delays timely treatment. These are possibly leading to severe neurological complications, including acute bilirubin encephalopathy, kernicterus, long-term developmental impairments, and sensorineural hearing loss [13–16]. Proper time for post-discharge follow-up of neonatal jaundice is important to prevent these serious consequences.

The American Academy of Pediatrics (AAP) 2009 guidelines had been used extensively to guide the management of neonatal hyperbilirubinemia in late preterm and term infants based on Bhutani nomogram which was based on bilirubin level, risk factors for severe hyperbilirubinemia and postnatal age [3]. Based on this guideline, the incidence of readmission in healthy neonates due to neonatal jaundice in Thailand is ranged from 3.8% to 24%. Most of these cases were infants who initially had no significant risk factors for severe jaundice [17–19].

In 2021, Kuzniewicz et al. compared different models for predicting the need for phototherapy after discharge. They found that using Δ-TSB (the difference between pre-discharge bilirubin level and the phototherapy threshold) was more accurate than the Bhutani Nomogram and may be easier to use in clinical practice [20]. AAP released new guidelines for managing neonatal jaundice in 2022, which use the difference between pre-discharge total bilirubin and the new phototherapy threshold (Delta TB), and with neurotoxicity risk factors to determine the follow up time [5]. Since its implementation, no study has evaluated the effectiveness of this recommendation in Thailand. To the best of our knowledge, this is the first study in Thailand to assess its application in clinical practice. The primary objective was to determine the proportion of post-discharge phototherapy after applying the new AAP 2022 guideline. The secondary objective was to evaluate the effectiveness of using pre-discharge DeltaTB to guide follow-up timing as a predictive tool for the need for post-discharge phototherapy.

## Method

This prospective cohort study was conducted at Chiang Mai University Hospital from August 2023 to December 2024. The study was approved by the Research Ethics Committee of the Faculty of Medicine at Chiang Mai University (Study code: PED-2566-0283). Neonates eligible for enrollment were inborn and healthy, with a gestational age of ≥ 35 weeks and a birth weight of ≥ 2000 g, and who had never received phototherapy before discharge. The exclusion criteria included newborns with direct hyperbilirubinemia or newborns requiring intensive care.

This study collected maternal and neonatal demographic data, and perinatal history from electronic medical record. Maternal data included age, gravidity, parity, gestational age (GA), blood group, underlying conditions, and antenatal care (ANC) laboratory results such as serology, thalassemia screening, and gestational diabetes mellitus (GDM) status. Perinatal history included the mode of delivery and any resuscitation during delivery. Neonatal data included gestational age, birth weight, sex, length, head circumference, discharge date, body weight before discharge and at follow-up time, scalp hematoma (e.g., cephalohematoma or subgaleal hematoma), neonatal sepsis, and feeding type (e.g., exclusive breastfeeding, formula, or both).

### Jaundice screening and follow up after discharge

At our institution, all healthy neonates are screened for jaundice by testing total serum bilirubin (TSB), direct bilirubin (DB) and hematocrit (Hct) at the age 48–72 h. They are typically discharged after 48 h of life and have at least one post-discharge follow-up visit. To streamline clinical practice, we categorized neonates into three risk groups based on their pre-discharge DeltaTB levels, with follow-up timing determined according to the 2022 AAP guidelines [5].

- High risk group: neonates with a DeltaTB of < 3.5 mg/dL, follow-up at 1 day after discharge.
- Moderate risk group: neonates with a DeltaTB 3.5–6.9 mg/dL, follow-up at 2 days after discharge.
- Low risk group: neonates with a DeltaTB value of > 7 mg/dL, follow-up at 3–5 days after discharge.

During the follow-up visit, medical history, vital signs, weight and physical examination findings were recorded. Blood sampling for TSB, DB and Hct was obtained. If the TSB level meets the phototherapy threshold according to the AAP 2022 guidelines, the neonate will be admitted for phototherapy and has the jaundice work up which includes complete blood count (CBC), reticulocyte count (RC), serum albumin, glucose-6-phosphate dehydrogenase (G6PD) level, blood group, indirect and direct antiglobulin test (IAT and DAT). The outpatient department discharge criteria for neonatal jaundice are either DeltaTB of > 7 mg/dL or appropriate weight gain. They are generally scheduled for subsequent follow-up visits until meeting the discharge criteria. Discharge criteria were TSB > 7 mg/dL below the phototherapy threshold, upward trend in body weight, and stable general condition.

### Sample size calculation

The sample size was calculated assuming a 95% confidence level, 5% margin of error, and 80% statistical power. Using an anticipated incidence of post-discharge phototherapy of 9.5% from Tantiprabha et al. [17], the minimum required sample size was 132 neonates. To account for an anticipated 10% dropout or loss to follow-up, the target sample size was increased to 150.

### Statistical analysis

Statistical analyses were conducted using IBM SPSS Statistics 25 (SPSS, Armonk, NY, USA). Continuous variables were described using the mean and standard deviation, depending on their distribution. Categorical data were analyzed using the Chi-square test. One-way ANOVA (Bonferroni) was used to compare the means outcomes between the three risk groups. DeltaTB levels in different age intervals were analyzed using ROC curve analysis to determine the cutoff in each interval.

To evaluate the diagnostic performance of DeltaTB at different age intervals in predicting post-discharge phototherapy, we calculated sensitivity, specificity, positive predictive value (PPV), negative predictive value (NPV), accuracy, and the area under the ROC curve (AUC) based on the optimal cutoff values.

### Definition

- Significant hyperbilirubinemia: A TSB level that is at or above the threshold for treatment, which varies based on the infant’s age in hours, risk factors, and gestational age.
- Subthreshold phototherapy: Phototherapy when TSB levels were 0.1 to 3.0 mg/dL below the appropriate AAP phototherapy threshold [21].
- Severe hyperbilirubinemia: TSB level above 25 mg/dL or within 2 mg/dL of exchange transfusion threshold [15].

## Results

**Fig. 1:**
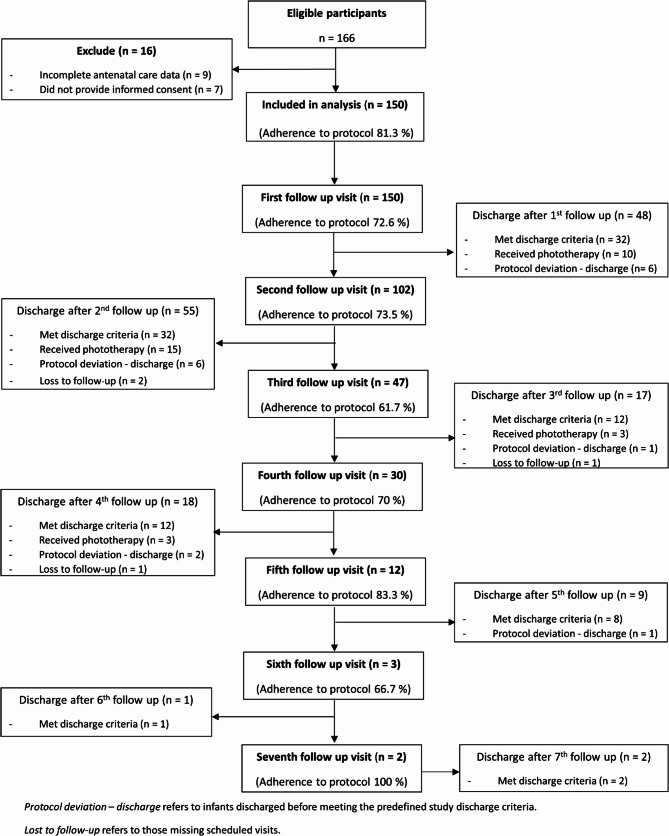
STROBE-compliant flow diagram of participant recruitment, follow-up visits, and discharge outcomes

Of 166 eligible neonates, 16 were excluded, leaving 150 for analysis. Follow-up visits and discharge outcomes at each visit are summarized in Fig. 1. Out of 150 neonates, 31 (20.6%) required post-discharge phototherapy. Of these, 22 (14.6%) neonates had significant hyperbilirubinemia, while 9 (6.0%) neonates received subthreshold phototherapy; no cases of severe hyperbilirubinemia were observed. The mean age onset of phototherapy was 115.87 ± 28.57 h. There were no neonates with severe hyperbilirubinemia. The mean GA was 38.25 ± 1.1 weeks, and the mean birth weight was 3,008 ± 389.8 g.

The most common cause jaundice in neonates who required post-discharge phototherapy was inconclusive jaundice (45%) followed by breast non-feeding (23%), scalp hematoma (13%), breastmilk jaundice (7%), ABO incompatibility (6%), G6PD deficiency (3%), and hemoglobin H disease (3%). Per our institutional protocol, all neonates required pre-discharge TSB and at least one post-discharge TSB at the follow-up visit. Among 150 neonates, 122 (81.3%) adhered to the follow-up protocol before discharge. The adherence decreased with subsequent follow-up appointments as shown in Fig. 1. The mean number of follow-up duration was 2.31 ± 1.34 times (range 1–7 times).

Table 1 shows demographic data comparing the post-discharge phototherapy and no phototherapy groups. Maternal factors, including advanced maternal age, nulliparity, gestation diabetes, maternal blood group O and positive thalassemia screening, were not significantly associated with post-discharge phototherapy. Similarly, perinatal factors including assisted vaginal delivery and resuscitation were also not significantly different. Scalp hematoma was more common in the phototherapy group (12.9% vs. 4.2%, *P* = 0.088) but not statistically significant. Pre-and post-discharge percentage of weight loss from the birth weight was higher in the phototherapy group, only post-discharge percentage of weight loss (−4.18% vs. −2.19%, *P* = 0.041) was statistically significant. Other neonatal factors, including male sex, small for gestational age, and exclusive breast feeding were not significantly different.

**Table 1:** Demographic data

Baseline characteristics	No Phototherapy (*n* = 119)	Phototherapy (*n* = 31)	*P*-value
Maternal factors			
• Advanced Maternal age	39 (32.7%)	6 (19.3%)	0.188
• Preterm	7 (5.9%)	3 (9.7%)	0.432
• Nulliparity	61 (51.3%)	18 (58.1%)	0.549
• Multiparity	62 (52.1%)	14 (45.2%)	0.548
• GDM	36 (30.3%)	8 (26.7%)	0.815
• Blood group O	46 (38.7%)	10 (32.3%)	0.830
• Positive thalassemia screening	1 (0.9%)	2 (6.7%)	0.112
Perinatal factors			
• Assisted Vaginal Delivery	8 (6.7%)	1 (3.2%)	0.686
• Resuscitation	9 (7.6%)	2 (6.5%)	1.000
Neonatal factors			
• Gestational age, weeks, Mean (SD)ZZZZ	38.3 (1.13)	37.9(1.0)	0.274
• Birth weight, grams, Mean (SD)	3,033 (401)	2,928 (347)	0.391
• Male	55 (46.2%)	16 (51.6%)	0.687
• SGA	13 (10.9%)	3 (9.7%)	1.00
• Scalp hematoma	5 (4.2%)	4 (12.9%)	0.088
• Presumed early onset neonatal sepsis	2 (1.7%)	0 (0%)	0.467
• Exclusive breast feeding	78 (65.5%)	23 (74.2%)	0.399
• Pre-discharge %weight loss, Mean (SD)	−1.32% (0.33)	−2.87% (0.9)	0.053
• Post-discharge %weight loss, Mean (SD)	−2.19% (0.44)	−4.18% (0.81)	0.041

**Fig. 2:**
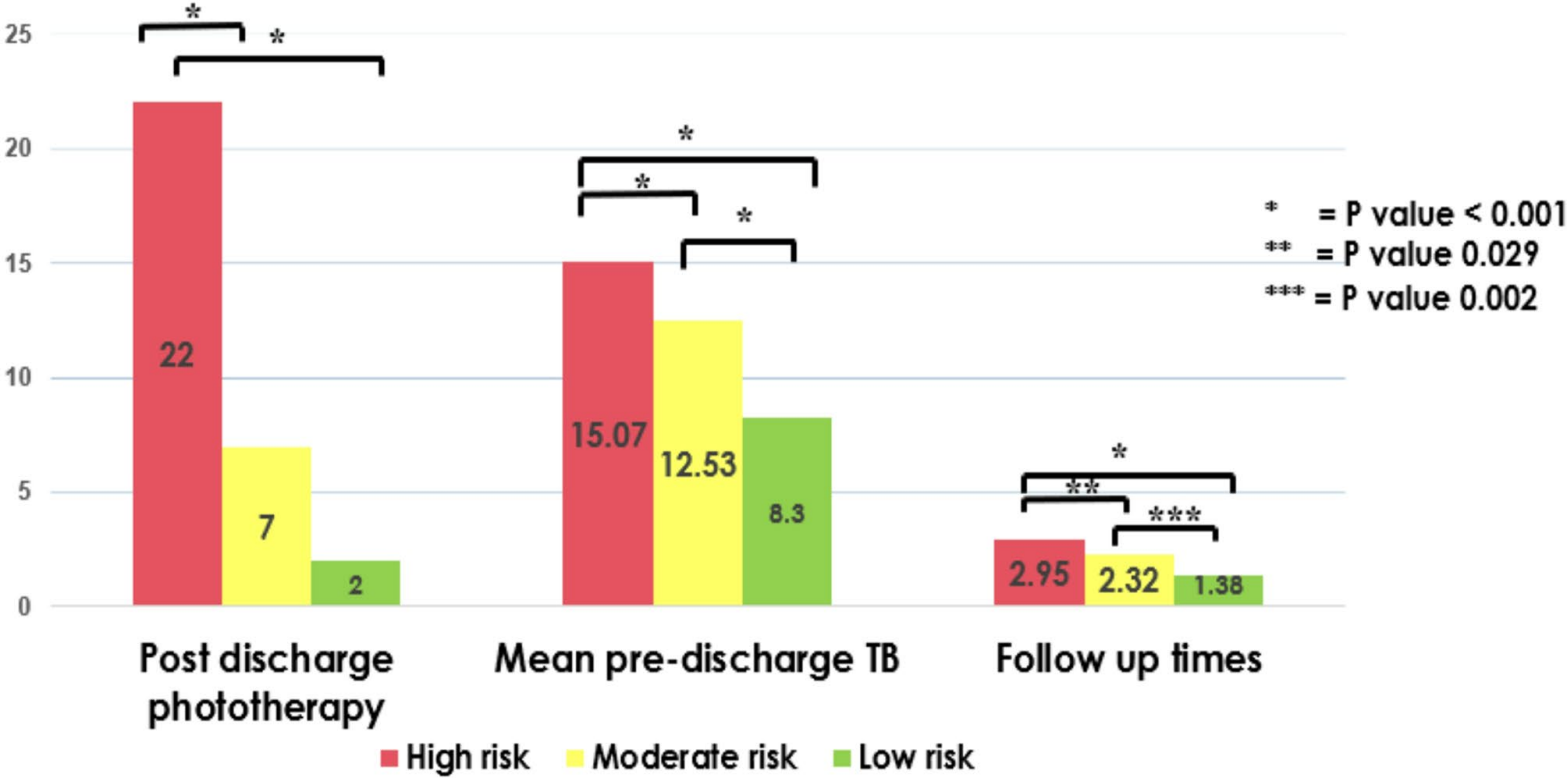
Comparison of incidence of post-discharge phototherapy, mean post-discharge TSB, and number of follow-up visits among risk groups categorized by pre-discharge DeltaTB

Based on their pre-discharge DeltaTB, 150 newborns were divided into three risk categories. There were 41 neonates (17.3%) in the high-risk group, 80 neonates (53.3%) in the moderate-risk group, and 29 neonates (19.3%) in the low-risk group. Figure 2 shows significant differences in outcomes across risk groups. Post-discharge phototherapy proportion was highest in the high-risk group (22 cases, 53.6%), significantly higher than in the moderate-risk (7 cases, 8.7%) and low-risk groups (2 cases,6.9%), with *P* < 0.001. Mean pre-discharge TB levels were also significantly higher in the high-risk group (15.07 ± 1.65 mg/dL) compared to the moderate-risk (12.53 ± 2.70 mg/dL) and low-risk groups (8.3 ± 4.57 mg/dL), with *P* < 0.001. Follow-up times were significantly higher in the high-risk group (2.95 ± 1.67 times) compared to moderate-risk (2.32 ± 1.12 times) with *P* = 0.029 and low-risk groups (1.38 ± 0.72 times) with *P* = 0.002. The optimal time for pre-discharge bilirubin screening was 49–72 h, with the highest area under the curve (AUC) at 0.851. At the cutoff level of 3.5 mg/dL at 49–72 h of life, sensitivity and specificity were 80% and 82% respectively. With different time intervals, the cutoff levels of DeltaTB for predicting post-discharge phototherapy were different. Table 2 demonstrates that the cutoff DeltaTB was higher with early pre-discharge bilirubin screening than for later screening.

**Table 2:** Predictive ability and cutoff of DeltaTB at different time intervals

Age (hours of life)	Area Under Curve (AUC)	Cutoff DeltaTB (mg/dL)	Sensitivity	Specificity	Positive predictive value (PPV)	Negative predictive value (NPV)
48	0.783	3.7	77%	76%	46.7%	92%
49–72	0.851	3.5	80%	82%	55.5%	93%
73–96	0.836	2.5	80%	77%	69%	60%

## Discussion

In this study, the proportion of neonates requiring post-discharge phototherapy was 20.6%, higher than previous studies conducted in Thailand, which ranged from 3.8% to 9.5% [17–19]. However, in the study by Kankaew et al. [22] conducted at Ramathibodi Hospital, the incidence was 24%, nearly the same in this study. Notably, the previous studies used the AAP 2009 phototherapy guidelines, and some institutions included subthreshold phototherapy during birth admissions, while this study uses the AAP 2022 phototherapy guidelines, and our institution did not have guidelines for subthreshold phototherapy during birth admission. Also, the research by Wickremasinghe et al. [21] showed that neonates who got subthreshold phototherapy during birth admission had a lower chance of being readmitted for post-discharge phototherapy without adverse outcomes. The study by Sarathy L et al. showed that the overall readmission rate for phototherapy remained stable after the new AAP 2022 guidelines (0.9% vs. 0.8%). However, among readmitted jaundice neonates, those exceeding the phototherapy threshold increased from 11% to 22.1%, and those receiving subthreshold phototherapy (exceed more than 2 mg/dL below the threshold) rose from 33% to 50.5% [23]. These findings suggest that while the overall rate of post-discharge phototherapy has remained stable, the characteristics of affected neonates have changed, with a higher reach of the phototherapy threshold and an increase in subthreshold phototherapy cases. This differs from our study, which observed a rising trend in the proportion of post-discharge phototherapy following the implementation of the new guidelines, along with an increase in subthreshold phototherapy cases. The higher rate of post-discharge phototherapy in this current study compared to previous studies could be due to the new phototherapy threshold changes based on the gestational age of neonates, and our hospital did not use subthreshold phototherapy guidelines. It may cause neonates who have low DeltaTB values, especially those who are in high-risk groups, to be discharged and have close follow-up within 1–2 days. At follow-up time after discharge, their bilirubin levels met the threshold for phototherapy.

In previous studies, the main causes of post-discharge phototherapy were inconclusive jaundice and breast non-feeding jaundice [17–19], which is consistent with our findings, where inconclusive jaundice was the leading cause, followed by breast non-feeding jaundice.

The significant risk factor for post-discharge phototherapy in our study was post-discharge % weight loss greater than 4.18% at first follow up. This finding was consistent with other studies, such as the one by Booranavanich et al. at Vajira Hospital [18], which reported that weight loss greater than 3.31% was associated with post-discharge phototherapy. Similarly, the study by Prachukthum et al. at Thammasat Hospital [19] show that weight loss of greater than 6% on the second day of life was a risk factor for post-discharge phototherapy. These results suggest that the higher percentage of weight loss in the early days of life is a significant factor influencing the need for post-discharge phototherapy.

This study found that the mean age onset of post-discharge phototherapy was 115 h, or Day of Life (DOL) 4 to 5. This finding was similar to the study by Prachukthum et al. at Thammasat Hospital [19], where the onset of post-discharge phototherapy occurred around DOL 6. These results are consistent with the natural progression of neonatal jaundice, as total bilirubin levels typically rise and peak approximately on DOL 3 to 5 (72–120 h of age) [6, 8].

In this study, DeltaTB levels were categorized into three risk groups: high risk (DeltaTB < 3.5), moderate risk (DeltaTB 3.5–6.9), and low risk (DeltaTB > 7). It was found that the proportion of post-discharge phototherapy was highest in the high-risk group as expected. However, even within the low-risk group, there remained a possibility of requiring post-discharge phototherapy. This highlights the importance of follow-up after discharge, as it is crucial for early detection and appropriate intervention, regardless of the initial risk classification.

The optimal time to screen pre-discharge total bilirubin is between 49 and 72 h, as this provides the best prediction for the likelihood of post-discharge phototherapy. A cut-off value of 3.5 is identified as the threshold for predicting the need for post-discharge phototherapy with sensitivity at 80% and specificity at 82%. Additionally, it was observed that the younger the infant’s age at the time of pre-discharge total bilirubin screening, the higher the cutoff value tends to be. This suggests that age at screening plays a role in determining the appropriate DeltaTB threshold for predicting the need for post-discharge phototherapy.

The strength of this study is that it was a prospective cohort study in which all neonates were followed at least once to check their TSB levels. Importantly, this is the first study in Thailand to determine the proportion of neonates requiring post-discharge phototherapy after implementation of the newly adopted AAP 2022 neonatal hyperbilirubinemia guideline and to assess the utility of DeltaTB in predicting this need. This study also has several limitations. First, as a single-center study, the findings may not be generalizable to other settings. Second, adherence to the follow-up protocol was approximately 80%. This reduced adherence may be due to this guideline used by various levels of healthcare providers, which could result in inconsistent protocol compliance and varied management practices. It would be beneficial to provide better training, streamline the protocol, and monitor across all levels of healthcare providers to improve adherence. Subthreshold phototherapy during newborn admission should be used in high-risk groups, as it may reduce the post-discharge phototherapy rate. Furthermore, future research should involve multi-center cohort studies for validation and focus on clinical implications, such as the cost-effectiveness of pre-discharge subthreshold phototherapy.

## Conclusion

In this first study in Thailand evaluating the new AAP 2022 neonatal hyperbilirubinemia guideline, 20.6% of neonates required post-discharge phototherapy. Neonates with a DeltaTB < 3.5 mg/dL had the highest risk, although some low-risk neonates also required treatment. These findings support the need for at least one post-discharge follow-up for all neonates to prevent delayed recognition and treatment of severe hyperbilirubinemia. DeltaTB-guided follow-up may facilitate risk-based identification, ensure timely post-discharge visits and interventions, and improve clinical outcomes.

## Data Availability

No datasets were generated or analysed during the current study.
